# Notes and News

**Published:** 1888-12-01

**Authors:** 


### Notes and News.

Collected and Communicated.

At the Charing Cross dinner last week, the President gave some interesting facts concerning royalty and charity, when proposing the health of the Queen. He reminded his hearers that the Charing Cross Hospital was the first institution to which Her Majesty lent her name, and which established a Victoria Ward. He also remarked that the Duke of Edinburgh was president of the Hospital, and had sent his good wishes and a donation of 25 guineas.

Hospital Sunday was celebrated at Romford last week, there being a large procession of various friendly societies of the town. Special services were held at all the churches, and about £80 was collected in aid of the Victoria Cottage Hospital. The amount collected in the streets was £26. The Hospital was opened in May last, and since then 26 patients have been received, of whom 20 were discharged cured, and one died.

At last it seems as though Sutton, Surrey, was to have its infectious hospital. At the last meeting of the Board of Guardians, Dr. Wilton proposed several sites, suggested that the hospital should consist of three small buildings with communication between them, that there should be six wards in all, with two beds in each ward, and that the cost should be about £50 a bed. It will be a fitting conclusion to his long and useful career at Sutton, if Dr. Wilton manages to start this hospital ere he retires into private life next spring.

The attitude taken by the Lord Mayor at the meeting of The Hospitals Association at Barts, shows that he is wishful to aid charities in the wisest manner. In introducing Sir Sydney Waterlow to the audience, the Lord Mayor stated that he had sat for some years at the feet of Gamaliel, and had drunk in words of wisdom. Mr. Alderman Whitehead is certainly a good friend to all the hospitals, as was proved by his bearing in the chair for the first time at the meeting of the Hospital Sunday Council on Monday last.

The first general meeting of the subscribers to the Faversham Cottage Hospital was held on November 16th. The hospital was only opened in May last, and since that date seventeen patients have been admitted. The hospital started under very favourable auspices; a site was presented by Messrs. Rigden, and Mrs. Harcourt Rose bore the building expenses. Under these circumstances it is not to be wondered at that the governors yet have a balance in hand, and are able to issue a satisfactory report.

The sanitation of the General Hospital at Birmingham is just now the theme of discussion in that town. The house surgeon is invalided with a sore throat, and it is alleged that no house surgeon has ever lived in the hospital who has not suffered from some form of blood-poisoning. It is also said that the rate of mortality in the female wards is very high, and that no difficult confinement case could be safely treated within their precincts. If things are really so bad as all this, it is quite time the committee took strong measures to improve the sanitation.

A "silver fete" has just been held at Dublin in connexion with the Children's Hospital in Harcourt Street. Two years ago the committee purchased the premises now in use at a cost of £3,000, and a debt of £1,200 still remains, to help to pay off which several friends of the hospital inaugurated a fancy fair. The proceedings were as pretty and as merry as such gatherings usually are, and the financial result is not yet known. For several months a few out of the fifty beds which the hospital holds have had to be kept empty, so as not to increase the weight of debt already such a burden to good work. We hope these beds can now be used.

The Bristol General Hospital had an unpleasant experience on November 22nd. A Jersey ketch loaded with 300 barrels of volatile naphtha, which was lying in the harbour just beneath the hospital, suddenly exploded, shattering every pane of glass in the building, to the number of about one thousand. There was a dreadful panic amongst the patients, and indeed there was imminent danger of the hospital catching fire for some time; but luckily the wind veered to the west, and the danger decreased. Four wards of the hospital have been closed, and it is stated that, but for the courage and coolness of the medical and nursing staff, the panic would have resulted in serious danger to the patients.

In a letter just addressed to Professor Virchow, president of the Children's Hospital in the north of Berlin, the Empress Frederick says: "It touched me deeply that the committee of a hospital to which I had only lately, with the sanction of my late Consort, the Emperor and King Frederick, been able to give my patronage, should have remembered the 18th of October—a day formerly welcomed with unmingled joy, but which cannot now but fill our hearts with mourning. In my deep grief, I return my thanks for this expression of consoling sympathy. It is a comforting and exalting feeling for me to be called to take an active part in a work of love in which the late Emperor and King felt so warm an interest."

The Home for Sick Children at Cold Ash, near Newbury, has lately been enlarged by the addition of a new ward to hold ten beds. The home was started two years ago by Miss Bowditch, who has found it so successful, thanks mainly to the bracing air of Cold Ash, that she has asked a committee to undertake the management of the enlarged building, while she devotes herself entirely to the work of lady superintendent. Several London hospitals send their semi-convalescent patients to Miss Bowditch; for instance, the Alexandra, Victoria, and Evelina hospitals; so that the present appeal for funds to help the new ward, which is specially for poor children, should be replied to, not only from neighbours of the home, but from the metropolis.

The recent atrocities at the East End have thrown a large amount of work on to the Missions for Friendless and Fallen Women. With the winter staring them in the face and their shelters besieged by an increasing crowd of wretched women and girls who have been roused to behold the horror of their existence, it is no wonder that these institutions are appealing for funds. The London Female Preventive and Reformatory Institution, which has its offices at 200, Euston Road, is for ever connected in the public mind with the name of Raikes, the founder of Sunday Schools. That Mr. Raikes, with the aid of Canon Dale, also founded this mission for fallen women is not so well known, though Mrs. Raikes acted as lady superintendent to the home till the day of her death. The mission has grown steadily of late years, and now accommodates 100 women and girls in the Preventive Homes, 94 in the Reformatory Homes, and 20 in the Open-All-Night Refuge at King's Cross. The society does not lose sight of those taken under its charge, and when possible helps them to emigrate or start a new life amidst new surroundings.

THE following vacancies are announced this week, the amount of salary in each case being given after the name of the institution :—

**Resident Medical Officers.**

London Throat Hospital, Great Portland Street. House Surgeon.
Chelsea Hospital for Women, Fulham. Three Clinical Assistants.
Royal Hants County Hospital. House Surgeon.—£100, board, etc.
Staffordshire General Infirmary. Assistant to House Surgeon.—Board.
North Eastern Hospital for Children. Junior House Surgeon.—£30.
The Samana and Santiago Railway Company. Medical Officer.—£200, residence, etc.
Bradford Borough Fever Hospital. Medical Superintendent.—£150, board, etc.
Holloway Sanatorium. Senior Assistant Officer.—£250.
Hospital for Insane, Virginia Water. Junior Assistant Medical Officer.—£120, board, etc.
Sunderland Infirmary. House Physician.—£80.
Westminster Hospital. Curator of Museum.—£40.

**Non-resident Medical Officers.**

St. George, Hanover Square, Provident Dispensary. Surgeon Dentist.
Birmingham Free Hospital for Sick Children. Acting Surgeon.
University of Edinburgh. Additional Examiner in Medical Jurisprudence.—£75.
Royal South London Dispensary. Surgeon.—£20.
London Temperance Hospital. Registrar and Chloroformist.—£50.
Free Hospital for Children, Kingsholm. Surgeon.

---

HOSPITAL SATURDAY has now become an almost universal institution, and we have lately been giving particulars of the amounts collected by its aid in different towns. Of course a great deal depends on the management of the collection, and the system commenced by Leeds last year appears to be the best. Instead of a procession, and one day in the year being solely devoted to the work, weekly collections of pennies and halfpence were begun, especially in the large manufactories. The result was a total sum of £3,700, whereas last year's total was only £2,000. The population of Leeds is about 334,000, so that this year more than £1 to every 100 inhabitants was collected. Many large towns might consider whether it would not be well for them to adopt this system of Hospital Saturday collections.

---

ON November 20th, the Most Rev. Dr. O'Dwyer, Bishop of Limerick, reopened the St. John's Hospital of that town. The hospital is a very old one, and a building of great historical interest : from its portals Ireton hanged his enemies, and the Branderburg regiment was blown to pieces. In 1832 it was used as a cholera hospital, and since then at intervals as a fever hospital. Not long ago a Catholic gentleman offered the Bishop of Limerick £1,500 if he would introduce the Sisters of the Little Company of Mary into his district. The Bishop thought of the disused St. John's Hospital, and applied to the Board of Governors, who gave him permission to improve and restore it, and hand it over to the Sisterhood. The opening ceremony was brief but hearty, and the visitors were conducted round the wards by the sisters.

---

AT the annual meeting of the Belfast Royal Hospital subscribers last week several interesting facts were made known. The total of in-patients amounted to 2,712, while the out-patients numbered over 13,000 in the year. This includes the patients in the children's hospital and in the consumptive department and convalescent home. Mr. McKenzie, in stating that the number of annual subscribers had diminished, said he thought that the hospital was losing favour in public opinion. This was emphatically denied by the Rev. Dr. Hanna and others, who pointed out that Hospital Sunday and the state of trade were responsible for fewer subscriptions. With regard to the late regretted case of accidental poisoning within the wards, it was stated that special three-corner cupboards were being made to hold all poisons, and that so far as human caution could prevent a recurrence of such an accident everything was being done.

THE story of the Tynemouth Infirmary, which lately held its thirteenth annual meeting, is an interesting one. In 1875 a number of young persons, who were in the habit of attending a Bible-class on Sunday afternoons, held in a school-room behind St. Andrew's Chapel, founded the "Flower Mission." The members visited the sick—carrying with them gifts of newly-cut flowers, singing hymns, and otherwise comforting the suffering. In the year 1877 this society acquired a new name. It became "The Benevolent Society and Flower Mission," which undertook the additional work of lending out sick-room requisites. On August 1st, 1884, the third stage of development was reached. The comprehensive title, "The Borough of Tynemouth Infirmary and Benevolent Society" was adopted. The fourth stage was reached on the occasion of Her Majesty's Jubilee, when the foundation-stone of "The Victoria Infirmary" was laid in the borough, and the happy goal will be gained next June, when the building is, according to present arrangements, to be opened and dedicated to public use for all time.

---

AT a meeting of the Council of the Hospital Sunday Fund held at the Mansion House, E.C., on Monday, the Lord Mayor, the Right Hon. James Whitehead, presiding, the following important resolution was unanimously agreed to, on the motion of Mr. Alfred L. Cohen, who is the head of the eminent firm of Messrs. Louis Cohen and Sons : "That it be a recommendation to the Council of 1889 that sub-committees be formed consisting of three leading members of the professions and the banking and commercial firms in London to canvass for donations amongst their own group or section, and that the results of these subscriptions be advertised under the respective headings of the contributing professions or businesses—for example, the Bankers, the Stock Exchange, and the Corn Exchange subscriptions. That the president and vice-president be empowered to nominate gentlemen to serve on these sub committees." The Rev. R. J. Simpson, Mr. Burdett, Sir Sydney Waterlow, Rev. J. F. Kitto, Dr. Hare, and others, spoke strongly in favour of Mr. Cohen's excellent proposal, which was unanimously approved.

---

WE welcome Mr. Cohen's action in this matter as full of promise and encouragement to metropolitan hospital managers of every degree. When a representative man of Mr. Cohen's high standing and position spontaneously takes up the cause of all the hospitals, and evolves a scheme which must bring their claims forcibly to the notice of very many wealthy people, who at present give little, if anything, to their support, the cause of the voluntary principle of medical charity must, indeed, be looking up. The late Canon Miller used to forcibly urge that giving to charities was as much a matter of education as the three R's. No one could realise the luxury and satisfaction to be gained from its cultivation until they had contributed liberally and often. Mr. Cohen's proposal is, not to attack individuals as individuals: that would be trenching too much on the proper functions of the collectors of each charity, but to approach them in their collective capacity, at the receipt of custom—*i.e*, in the Clearing House, and at the Baltic, Corn, Coal, Hop, Leather, Metal, Royal, Stock, Wool, and other Exchanges, including, of course, Lloyd's, where the fortunes are made in business, and from whence no contributions have yet been received year by year for all the hospitals, through the Lord Mayor as President of the Hospital Sunday Fund. This movement may have very important results; it could only succeed when individual and influential members of each Exchange agreed to actively interest themselves on behalf of all the hospitals amongst the members of their own markets, and the customs of these Exchanges renders it impossible to make an ordinary appeal for any single hospital or kindred institution. It will be recognised, then, that Mr. Cohen proposes to open up new ground entirely, and to do so in the only way by which success can be secured. All the hospitals will therefore feel indebted to Mr. Alfred Cohen for his kindly and powerful intervention in their behalf. We estimate that at least £10,000 a-year ought to be added to the Fund as a direct result of this new movement.

